# Supplementary material for: Multimorbidity gender patterns in hospitalized elderly patients
Source: PLoS One. 2020 Jan 28;15(1):e0227252. doi: 10.1371/journal.pone.0227252 (PMC6986758; doi:10.1371/journal.pone.0227252)
Supplement: S1 Table — (DOCX) [file pone.0227252.s001.docx]

Table S1. Multimorbidity criteria Spanish Health Department
