# Supplementary material for: Multimorbidity gender patterns in hospitalized elderly patients
Source: PLoS One. 2020 Jan 28;15(1):e0227252. doi: 10.1371/journal.pone.0227252 (PMC6986758; doi:10.1371/journal.pone.0227252)
Supplement: S5 Table — (DOCX) [file pone.0227252.s005.docx]

Table S-5

|  | Included (n=843) | Excluded (n=42) | p |
| --- | --- | --- | --- |
| Age | 82.4 (9.8) | 62.3 (19.1) | <0.0001 |
| Charlson index | 4.9 (3.4) | 0.1 (0.6) | <0.0001 |
| Barthel index | 49.4 (34.4) | 74.9 (38.6) | <0.0001 |
| Multimorbidity criteria | 3.2 (1.4) | 0.59 (0.5) | <0.0001 |
| Pfeiffer index | 4.2 (5.5) | 1.9 (3.5) | =0.008 |
| Length of stay (days) | 11.1 (8.8) | 9.1 (11.3) | =0.2 |
| Gender |  |  |  |
| Male | 415 (46.9%) | 24 (2.7%) | =0.3 |
| Female | 428 (48.4%) | 18 (2%) |  |
