# Supplementary material for: Multimorbidity gender patterns in hospitalized elderly patients
Source: PLoS One. 2020 Jan 28;15(1):e0227252. doi: 10.1371/journal.pone.0227252 (PMC6986758; doi:10.1371/journal.pone.0227252)
Supplement: S6 Table — (DOCX) [file pone.0227252.s006.docx]

Table S-6

|  | Men | Women | p |
| --- | --- | --- | --- |
| cardiovascular +respiratory | 179 (43.1%) | 161 (37.6%) | 0.1 |
| cardiovascular + metabolic | 281 (67.7%) | 295 (68.9%) | 0.3 |
| cardiovascular + neurologic | 176 (42.4%) | 219 (51.2%) | 0.1 |
| cardiovascular + osteoarticular | 53 (12.8%) | 145 (33.9%) | <0.0001 |
| cardiovascular + miscellanea | 161 (38.8%) | 176 (41.1%) | 0.5 |
| cardiovaculra + neoplasm | 45 (10.8%) | 22 (5.1%) | 0.002 |
| respiratory + metabolic | 201 (48.4%) | 174 (40.7) | 0.03 |
| respiratory + neurologicic | 127 (30.6%) | 123 (28.7%) | 0.6 |
| respiratory + osteoarticular | 39 (9.4%) | 86 (20.1%) | <0.0001 |
| respiratory + miscellanea | 115 (27.7%) | 103 (24.1%) | 0.2 |
| respiratory + neoplasm | 37 (8.9%) | 13 (3%) | <0.0001 |
| metabolic + neurologic | 228 (45.9%) | 276 (46.5%) | 0.005 |
| metabolic + osteoarticular | 69 (16.6%) | 179 (41.8%) | <0.00001 |
| metabolic + miscellanea | 189 (45.5%) | 205 (47.9%) | 0.5 |
| metabolic + neoplasm | 58 (14%) | 26 (6.1%) | <0.0001 |
| neurologic +osteoarticular | 55 (13.3%) | 156 (36.4%) | <0.0001 |
| neurologic + miscellanea | 128 (30.8%) | 158 (36.9%) | 0.7 |
| neurologic + neoplasm | 35 (8.4%) | 19 (4.4%) | 0.02 |
| osteoarticular + miscellanea | 43 (10.4%) | 114 (26.6%) | <0.0001 |
| osteoarticular + neoplasm | 9 (2.2%) | 8 (1.9) | 0.8 |
| miscellanea + neoplasm | 41 (9.9%) | 19 (4.4%) | 0.003 |
