# Supplementary material for: Multimorbidity gender patterns in hospitalized elderly patients
Source: PLoS One. 2020 Jan 28;15(1):e0227252. doi: 10.1371/journal.pone.0227252 (PMC6986758; doi:10.1371/journal.pone.0227252)
Supplement: Supplementary file 6 [file pone.0227252.s003.docx]

S3 Table.

PROFUND SCORE	points
>84 years	3
Active neoplasm	6
Dementia (Pfeiffer >5)	6
III-IV NYHA	3
3-3 mMRC	3
Delirium during admission	3
Hb<10	3
Barthel <60	4
No caregiver or caregiver other than partner	2
4 hospitalizations in the previous year	3
TOTAL	
